# Total Coumarins from *Hydrangea paniculata* Show Renal Protective Effects in Lipopolysaccharide-Induced Acute Kidney Injury via Anti-inflammatory and Antioxidant Activities

**DOI:** 10.3389/fphar.2017.00872

**Published:** 2017-12-14

**Authors:** Sen Zhang, Jie Ma, Li Sheng, Dongming Zhang, Xiaoguang Chen, Jingzhi Yang, Dongjie Wang

**Affiliations:** State Key Laboratory of Bioactive Substances and Functions of Natural Medicines, Department of Pharmacology, Institute of Materia Medica, Chinese Academy of Medical Sciences & Peking Union Medical College, Beijing, China

**Keywords:** sepsis, acute kidney injury, coumarin, *H. paniculata*, pharmacokinetics, anti-inflammation, NF-κB, network pharmacology

## Abstract

**Background:** Septic acute kidney injury (AKI) causes high mortality in critical care units, and no effective therapy exists in clinical treatment. In the current study, water and ethanol extracts of *Hydrangea paniculata* (HP), a traditional Chinese medicinal plant, were used to test its renoprotective effects in a lipopolysaccharide (LPS)-induced murine model of septic AKI.

**Methods:** C57BL/6 mice were orally pretreated with HP three times, and then intraperitoneal LPS injection was used to induce septic AKI. Blood from animals was collected for biochemical analysis and kidneys were obtained for pathological analysis. Kidney tissue homogenates were used to investigate the effect of HP on inflammation and oxidative stress. Immunohistochemistry was used to investigate tubular cell apoptosis. Flow cytometry was conducted to analyze leukocyte infiltration into the kidneys. Blood cell counts were used to analyze changes in peripheral leukocytes. *In vitro* studies with Ana1 and HK-2 cells stimulated by LPS were used to investigate the anti-inflammatory effects and inhibition of signaling pathways by HP.

**Results:** HP significantly decreased blood urea nitrogen and plasma neutrophil gelatinase-associated lipocalin concentrations, as well as tubulointerstitium injuries in septic AKI mice. Moreover, HP administration improved animal survival following lethal LPS injections. HP ameliorated apoptosis of tubular cells by inhibiting the cleavage of caspase 3 and caspase 7. HP also showed pronounced antioxidant activity in AKI kidneys. HP showed anti-inflammatory effects by inhibiting the infiltration of neutrophils and macrophages into kidney tissues induced by LPS, as well as inhibiting the production of cytokines and chemokines. Possible molecular mechanisms included HP inhibition of NF-κB nuclear translocation in LPS-induced macrophages and tubular cells, and reduction of STAT3, STAT1, and ERK1/2 phosphorylation stimulated by LPS *in vitro*. Single acute toxicity tests confirmed that HP, even at 5 g/kg dosage, does not cause animal death. Pharmacokinetics also showed that coumarins from HP could be metabolized into two bioactive compounds, umbelliferone, and esculetin.

**Conclusions:** HP extract may protect renal function in LPS-induced AKI by anti-inflammatory and antioxidant activities, and has potential in the critical care of AKI.

## Introduction

Sepsis is a disseminated inflammatory response elicited by a microbial infection (Holthoff et al., 2012) and is the major cause of death among intensive care unit (ICU) patients (Hotchkiss and Karl, 2003; Azevedo, 2013; Alobaidi et al., 2015). Unfortunately, there is no effective treatment for septic patients thus far (O'Neill et al., 2012). Development of acute kidney injury (AKI) is common during severe sepsis and significantly increases the patient mortality rate to nearly 75% (Heemskerk et al., 2009). Therefore, novel therapeutic interventions are urgently needed to manage this dangerous condition.

The knowledge of the pathophysiology of septic AKI has increased quickly in the past few years, and some traditional theory has been updated. Hemodynamic alteration was considered a major mechanism of AKI, and hypoperfusion was believed to reduce both glomerular filtration and cellular high-energy phosphates, finally producing acute tubular necrosis and apoptosis (Badr et al., 1986; Wan et al., 2008). However, new evidence does not support this theory and suggests that ischemia and bioenergetics failure likely represent only part of the mechanism responsible for loss of renal function. Therefore, there are multiple mechanisms working together that lead to deterioration in septic AKI (May et al., 2005, 2007).

Overproduction of inflammatory mediators, such as tumor necrosis factor-α (TNF-α), nuclear factor-κB (NF-κB), monocyte chemoattractant protein-1 (MCP-1), interleukin (IL)-6, and IL-1β, is considered to play an important role in the immunopathogenesis of septic acute kidney dysfunction, which results in kidney damage (Chen et al., 2008). Overexpression of cytokines and chemokines facilitates the migration, infiltration, and accumulation of inflammatory cells, such as macrophages and neutrophils, into tissues, which increases the release of cytotoxic reactive oxygen species, causing tissue damage (Yang et al., 2014; Kim et al., 2015). Controlling proinflammatory mediators could be an effective approach to treat kidney inflammatory injury (Chen et al., 2014). Activation of extracellular signal-regulated kinase (ERK) and p38 mitogen-activated protein (MAP) kinase (Mi Jeong et al., 2009), the Janus kinase/signal transducers and activators of transcription (Jak-Stat) pathway, and NF-κB/inhibitor of NF-κB (IκB) signal transduction pathways (Lu et al., 2008), were reported to play important roles in proinflammatory responses during sepsis.

Based on documents of traditional Chinese medicine, many medicinal plants show promising anti-inflammatory and anti-oxidative activities. *Hydrangea paniculata* Sieb is a medical herb, which is widely distributed in southern of China. It has been used to treat inflammation, malaria, and fever throughout Chinese history. Water and 20% ethanol extracts of *H. paniculata* (HP) provided by our institute contain high amounts of coumarin-glycosides; among them, skimmin and apiosylskimmin are major constituents, along with 10 other minor coumarin glycosides. Previous studies from our laboratory demonstrated that skimmin administration could slow the progression of streptozotocin-induced diabetic nephropathy (Zhang et al., 2012) and cationized BSA-induced membranous glomerulitis (Zhang et al., 2013), and that HP extract showed renal protective effects on cisplatin-induced AKI (Sen et al., 2017). Other coumarin glycosides from HP have shown hepatoprotective activities against DL-galactosamine-induced toxicity in HL-7702 cells (Shi et al., 2014), and neuroprotective effects against serum deprivation-induced PC12 cell damage (Shi et al., 2012).

Considering the traditional medical usage of HP and its bioactive ingredients, we hypothesized that HP extract would be beneficial against septic AKI, due to its antioxidant and anti-inflammatory activities, especially its specific renoprotective effect. The current study is designed to investigate the potential protective effect of HP extract, against renal injury caused by LPS, and to study its underlying mechanisms. Due to the complexity of the composition of HP, network pharmacology was also performed to predict the possible drug targets of its major constituents.

## Materials and methods

### Preparation of extract of *H. paniculata*

The stems of *H. paniculata* Sieb. (Saxifragaceae) were collected in the County of Jinxiu, Guangxi Zhuang Autonomous Region, China, in May 2016 and identified by Mr. Guangri Long (Liuzhou Forestry Bureau of Guangxi). A voucher specimen (ID-4645) was deposited at the Institute of *Materia Medica*, Chinese Academy of Medical Sciences, Beijing.

The preparation procedure of HP and its major constituents is described in a previous publication from our laboratory (Sen et al., 2017). To standardize the HP extract for each batch, the content of total coumarin glycosides was verified to be 70–75% by ultraviolet-visible (UV-Vis) spectroscopy; the two major ingredients, skimmin and apiosylskimmin, were verified to be 50–55%. The High-performance liquid chromatography (HPLC) profile of HP is shown in Supplemental Figure 1A. HP was dissolved in 0.5% sodium carboxymethyl cellulose (CMC-Na) for animal administration.

### LPS injection and mouse model of septic shock

**Figure 1:**
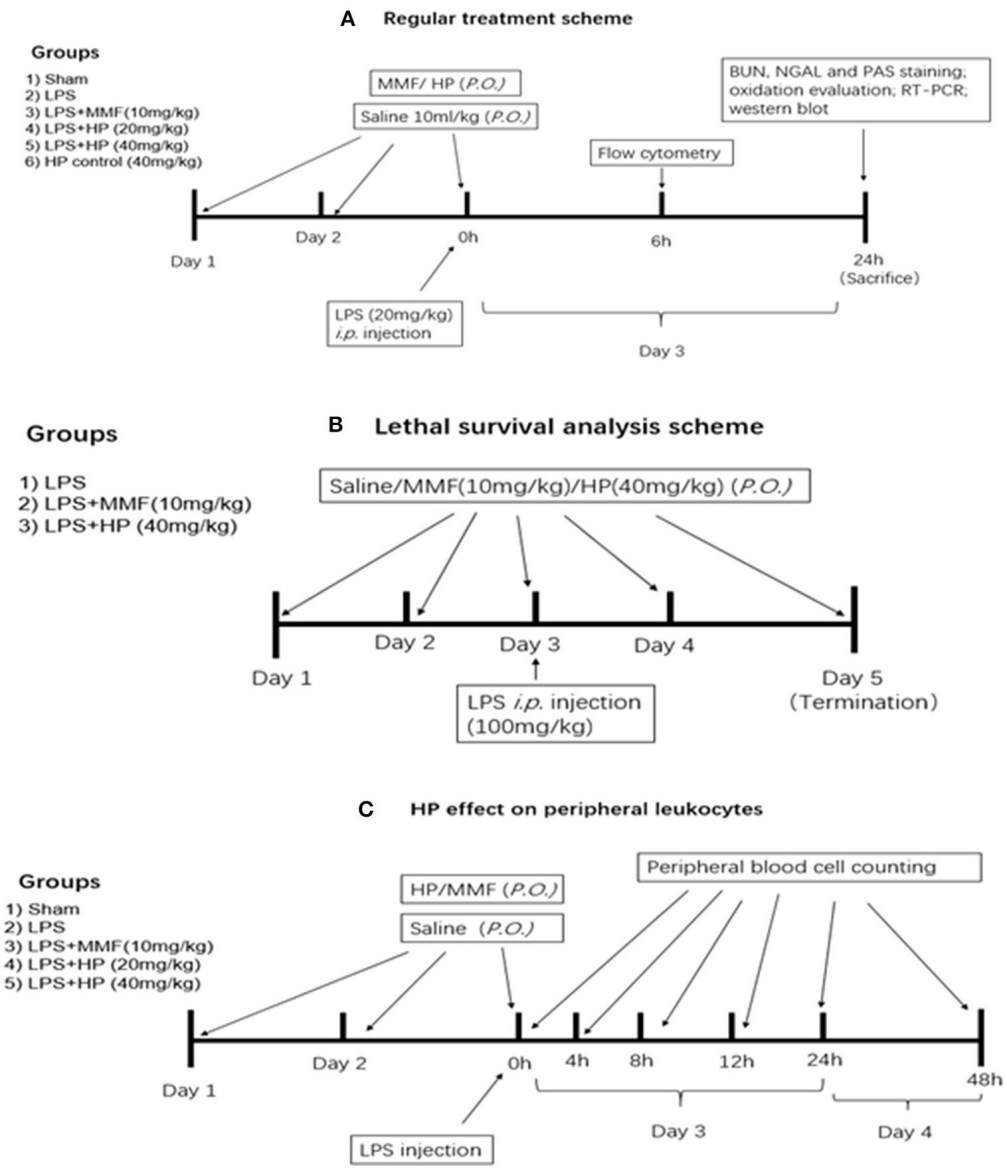
Experimental design, procedure, and animal group classification for the HP effect on LPS-induced acute kidney injury and survival analysis. **(A)** HP renal protective effect on AKI with regular LPS dosage; **(B)** survival analysis with LPS lethal dosage; **(C)** blood cell counting in peripheral blood.

LPS from *Escherichia coli* 0111:B4 was purchased from Sigma-Aldrich (St. Louis, MO, USA), and freshly dissolved in sterile 0.9% NaCl solution buffer before i.p. injection. For the standard treatment experiment, low dosage of LPS was used for induction of kidney injury. Inbred, 16–18 g, 4–6-week-old, male C57BL/6 mice were purchased from the SPF Biotechnology Co. Ltd. (Beijing, China). Six groups of mice were examined (*N* = 8 per group) and a detailed experimental procedure is shown in Figure 1A. The sham group received 10 mL/kg saline by oral gavagedaily during the experiment, and at day 3 received a saline sham injection (i.p.). The LPS model group was exposed to the same procedure as the sham group, except receiving an LPS (20 mg/kg) injection (i.p.) at day 3, instead of saline. The HP plus LPS groups received an oral pretreatment of HP at 20 or 40 mg/kg/day for 2 days prior to LPS injection on day 3; immediately after LPS injection, HP was given orally for the third time. Mycophenolate mofetil (MMF) was used as a positive control in the current study, with the MMF plus LPS group receiving 10 mg/kg MMF instead of HF. The HP group only received HP administration orally (40 mg/kg) three times.

Twenty-four hours after LPS injection, blood was collected from the endocanthion and all mice were euthanized by cervical dislocation. The kidneys were removed, fixed in 10% formalin, and embedded in paraffin for pathological investigation.

A survival study was performed with a lethal dosage of LPS, and the detailed experimental procedure is shown in Figure 1B. Briefly, three groups (*N* = 9 per group) were involved, which were the vehicle-treated control, HP (40 mg/kg)-treated, and MMF (10 mg/kg) positive control groups. After two consecutive days of vehicle/HP/MMF administration, all of the animals were injected intraperitoneally with a lethal dose of LPS (100 mg/kg). To determine the survival rate, the mice were observed for 48 h after LPS injection, and the exact time of death for each mouse was recorded.

This study was carried out in accordance with the recommendations of “Chinese guidelines for Proper Conduct of Animal Experiments, Ethics Committee of Laboratory Animals of Beijing Municipality.” The protocol was approved by the “Ethics Committee of Laboratory Animals of Beijing Municipality.” The approval number was 790826.

### Cell culture and cell viability assay

Human kidney-2 (HK-2) cells, an immortalized human proximal tubular cell line, were purchased from American Type Culture Collection (ATCC, Manassas, VA, USA); the murine macrophage Ana1 cell line was purchased from the National Infrastructure of Cell Line Resource (Beijing, China). The cells were grown in DMEM (Invitrogen, Paisley, UK) supplemented with 10% fetal bovine serum (FBS, Hyclone, Australia) for fewer than 10 passages. The growth medium was supplemented with 2 mmol/L glutamine, 100 U/mL penicillin, and 100 U/mL streptomycin, and the cells were maintained in a 37°C, 5% CO_2_, humidified atmosphere. Cultures were given fresh media every 2–3 days. Cells were incubated with various concentrations of HP (100, 50, 25, or 12.5 μg/mL) for 24 or 72 h. For cell viability assays, cells were cultured in 96-well plates, Cell Counting Kit-8 (CCK-8) was added into each well, cells were cultured for an additional 2 h, and the absorbance at 450 nm was measured. The cell viability inhibition (%) was calculated by the following formula: (control A_450_ − treated well A_450_)/control A_450_ × 100%.

### Measurement of biochemical index of renal functions in serum

Blood urea nitrogen (BUN) and neutrophil gelatinase-associated lipocalin (NGAL) were used as biomarkers to estimate early renal injury in the current study. The concentration of BUN was measured by a commercial BUN assay kit (Nanjing Jiancheng Bioengineer Institute, Nanjing, China), and NGAL was measured by an NGAL ELISA kit (R&D Systems, USA). Experimental procedures in the provided instruction manuals were strictly followed.

### Histology

Periodic acid–Schiff-stained sections were scored in a blinded, semiquantitative manner. For each animal, at least 10 high-power (×400) fields were examined. The percentage of tubules that displayed cellular necrosis, loss of brush border, cast formation, vacuolization, and tubule dilation was scored as follows: 0 (none), 1 (<10%), 2 (11–25%), 3 (26–45%), 4 (46–75%), and 5 (>76%).

### Measurement of oxidative stress markers *in vivo* and *in vitro*

Kidneys were homogenized in Phosphate-buffered saline (PBS) using a TissueLyser-38 (Jingxin Industrial Development Co. Ltd., Shanghai, China) and then centrifuged at 3,000 g for 10 min. The pellet was discarded and protein content was measured in the supernatant using a bicinchoninic acid (BCA) assay (Applygen Technologies Inc., Beijing, China). Supernatant aliquots were used to determine oxidative markers, including nitric oxide (NO), reduced glutathione (GSH), lipid peroxidation (LPO), superoxide dismutase (SOD) activity, and catalase (CAT) activity. The test procedure supplied with each commercial kit was followed (Nanjing Jiancheng Biotechnology, Nanjing, China).

### Immunohistochemistry analysis

For cleaved caspase 3 immunohistochemical analysis, the endogenous peroxidase activity in each section was quenched with 3% H_2_O_2_. Antigen retrieval was performed by heating in citrate buffer (PH = 7.0). Sections were incubated with primary antibody (rabbit monoclonal cleaved caspase 3, 1:100) overnight at 4°C. Labelling was detected with a biotin-conjugated goat anti-rabbit IgG and avidin-biotin peroxidase complex. Cleaved caspase-3-positive cells in cortexes were scored in 30 random × 400 high-power fields (HPFs) with a ZEISS Observer A1 microscope (ZEISS, Germany).

### Quantitative reverse transcription PCR (RT-qPCR)

The mRNA levels of selected important chemokines and cytokines in the kidneys, such as TNF-α, IL-1β, IL-6, intercellular adhesion molecule 1 (ICAM-1), vascular cell adhesion molecule 1 (VCAM-1), and MCP-1, as well as caspase family members, such as caspase 3, caspase 7, caspase 8, and caspase 9, were quantified by RT-qPCR. Briefly, total RNA was extracted from kidney tissues using TRIzol® (Life Technologies, Inc., USA), according to the manufacturer's instructions; the RNA (10 μg) from each group was reverse-transcribed by a reverse transcription kit (ReverTra Ace® qPCR RT Kit, Toyobo Inc., Japan) to obtain the corresponding cDNA. RT-qPCR was performed using the THUNDERBIRD® qPCR Mix (Toyobo Inc., Japan) on an ABI Prism 7900 sequence detection system (Applied Biosystems, CA, USA), and ΔCt was normalized to the signal of the housekeeping gene, GAPDH. The fold change of expression was calculated as 2^ΔCt^ (Treated − Untreated). The primer sequences are listed in Table 1. The qPCR cycle conditions were: one cycle of 95°C for 30 s, 40 cycles of 95°C for 15 s, 60°C for 30 s, and 72°C for 30 s.

**Table 1:** The primer sequences of murine TNF-α, IL-1β, IL-6, MCP-1, ICAM-1, VCAM-1, caspase 3, caspase 7, caspase 8, caspase 9, and GAPDH.

**Gene**	**Primer sequence (from 5′ to 3′)**
IL-1β forward	CACCTTCTTTTCCTTCATCTTTG
IL-1β reverse	GTCGTTGCTTGTCTCTCCTTGTA
IL-6 forward	CAAAGCCAGAGTCCTTCAGAG
IL-6 reverse	GCCACTCCTTCTGTGACTCC
TNF-α forward	CCCAGACCCTCACACTCCAGAT
TNF-α reverse	TTGTCCCTTGAAGAGAACCTG
MCP-1 forward	TTAAAAACCTGGATCGGAACCAA
MCP-1 reverse	GCATTAGCTTCAGATTTACGGGT
ICAM-1 forward	CAATTTCTCATGCCGCACAG
ICAM-1 reverse	AGCTGGAAGATCGAAAGTCCG
VCAM-1 forward	TGAACCCAAACAGAGGCAGAGT
VCAM-1 reverse	GGTATCCCATCACTTGAGCAGG
Caspase 3 forward	TGACTGGAA AGCCGA AACTC
Caspase 3 reverse	AGCCTCCACCGGTATCTTCT
Caspase 7 forward	GGACCGAGTGCCCACTTATC
Caspase 7 reverse	TCGCTTTGTCGAAGTTCTTGTT
Caspase 8 forward	CCG AGCTGG ACTTGTGACC
Caspase 8 reverse	CTGCCCAGTTCTTCAGCA AT
Caspase 9 forward	AGGAGGGACGAACACGTCT
Caspase 9 reverse	CAAAGAAGGTTGCCCCAATCT
GAPDH forward	CGACTTCAACAGCAACTCCCACTCTTCC
GAPDH reverse	TGGGTGGTCCAGGGTTTCTTACTCCTT

### Sub-group distributions of immune-related cells in mouse peripheral blood

To understand the effect of HP on peripheral leukocytes in LPS-induced septic mice, peripheral plasma was collected in heparin pretreated tubes by retro-orbital bleeding at 4, 8, 12, 24, and 48 h post-LPS injection, and the number of monocytes, lymphocytes, and neutrophils in the plasma at different time points was examined by an Automatic Hematology Analyzer (Celltac E MEK-7222, Nihon Kohden, Japan). The detailed experimental procedure is shown in Figure 1C, which is similar to that used to analyze the effect of HP treatment on LPS-induced AKI.

### Flow cytometric determination of kidney leukocytes

To determine the effect of HP on leukocyte populations in septic kidneys, kidney single-cell suspensions were prepared and analyzed. Briefly, the single-cell suspensions were first incubated with anti-mouse CD45-APC-Cy7 antibody (eBioscience, USA) for 30 min on ice. After washing, the cells were stained for 15 min on ice with fluorochrome-labeled monoclonal antibodies against anti-mouse Ly6G-PE, CD11c-FITC, or F4/80-APC (eBioscience, USA) for detection of neutrophils or macrophages. Six-color fluorescence flow cytometric analyses were performed (FACS Verse, BD, USA), and the data were analyzed with the FlowJo program (Tree Star, Inc., Ashland, CA, USA).

### HP effects on LPS-induced proinflammatory signaling pathways

Ana1 and HK-2 cells were pretreated with HP at various concentrations for 2 h before LPS (50 μg/mL) was added. Cells were collected at different time points after LPS addition (2, 4, 8, 12, and 24 h), and total protein extracts, or nuclear and cytoplasmic protein extracts were prepared. NF-κB nuclear translocation and phosphorylation of STAT1, STAT3, and ERK1/2 in cells were examined, as indicated below.

### Cytosolic and nuclear extraction and NF-κB nuclear translocation assessment

Ana1 cells and HK-2 cells, which were prepared by the abovementioned procedure, were subjected to cytosolic and nuclear protein extraction using the NE-PER™ Nuclear and Cytoplasmic Extraction Kit (Thermo Fisher Scientific, NY, USA). Briefly, Cytoplasmic Extraction Reagent I (provided in the kit) was added to the cell pellets, and, after 15 min incubation on ice, the homogenates were centrifuged at 13,000 g for 1 min at 4°C. The supernatants (cytoplasmic extracts) were stored at −80°C. The nuclear pellets were resuspended in Nuclear Extraction Reagent (provided in the kit), and the tubes were vigorously rocked at 4°C for 30 min on a shaking platform. The tubes were then centrifuged at 13,000 g for 15 min at 4°C. The supernatants (nuclear extracts) were frozen in aliquots at −80°C until use. Protein content was determined by the BCA method. The assessment of NF-κB nuclear translocation was made by western blot.

### Western blot

Proteins from cytoplasmic and nuclear fractions were added to 2 × sodium dodecyl sulfate (SDS) sample buffer [0.125 M Tris-HCl (pH 6.8), 4% SDS, 20% glycerol, 10% 2-mercaptoethanol, 0.004% bromophenol blue], and boiled in a water bath for 5 min. Protein samples (100 μg per lane) were separated on 10% SDS polyacrylamide gels and transferred to nitrocellulose membranes. Non-specific binding to the membrane was blocked for 1 h at room temperature with 5% nonfat milk in Tris-buffered saline with 0.1% Tween® 20 (TBS/T). Membranes were then incubated at 4°C overnight with primary antibody against nuclear NF-κB p65 (1:1,000) or cytosolic IκB-α (1:500) in 1% TBS/T containing 5% nonfat milk. The membranes were washed three times with 0.1% TBS/T, and then incubated for 1 h at room temperature with a secondary antibody (anti-rabbit IgG, peroxidase-conjugated). Lamin B1 and β-actin were used as internal standards for nuclear and cytosolic extracts, respectively. The immunoreactive bands were visualized using an enhanced chemiluminescence system (LAS 4000, GE healthcare, CA, USA). The same western blot procedure was performed on ERK1/2, p-ERK1/2, STAT1, p-STAT1, STAT3, and p-STAT3 (Cell Signaling Technology, CA, USA).

### Pharmacokinetics study

The male C57BL/6 mice were divided into several subgroups at different time points to collect blood samples (*N* = 5 for each subgroup). All of the animals were fasted for 12 h before receiving HP and were then allowed to eat for 4 h after administration. The dosing solutions used for the animal studies were prepared by dissolving the required amounts of HP in distilled water. After oral administration of HP at a dose of 50 mg/kg to the mice, approximately 50 μL blood was collected in heparinized 1.5 mL polyethylene tubes by retro-orbital bleeding via capillary tubes at 0.08, 0.25, 0.5, 1, 2, 4, 6, 8, 12, 24, 36, and 48 h post-dose. The blood samples were immediately centrifuged at 5,000 × g for 10 min. The plasma was separated and stored at 2–8°C until analysis. For determination of skimmin, apiosylskimmin, and their predicted metabolites (umbelliferone and esculetin), each plasma sample (15 μL) was spiked with 20 μL of all four internal standards (500 ng/mL), purchased from Sigma-Aldrich (St. Louis, MO, USA) and then mixed with 180 μL of acetonitrile (Merck, Darmstadt, Germany) to precipitate protein. After centrifugation, aliquots (10 μL each) of the supernatant were used for analysis. Skimmin and apiosylskimmin were determined using liquid chromatography–MS/MS, consisting of an Agilent 1260 Series high performance liquid chromatography system, fitted with a Zorbax SB-C18 analytical column (3.5 mm, 100 mm, 2.1 mm, Agilent Technologies, Santa Clara, USA) and an API 4000 triple quadruple mass spectrometer (AB SCIEX, USA). The mobile phase consisted of solvent A [0.1% formic acid (Dikma Technologies, Inc., CA, USA) in water] and solvent B [0.1% formic acid in methanol (Merck)]. The analytes were eluted at a flow rate of 0.2 mL/min, with the following gradient elution: from 0 to 0.6 min, 20% B; linear increase to 95% B in 0.2 min; 95% B for 4 min, decrease to 20% B in 0.2 min; and stabilization at initial conditions for 6 min. The mass spectrometer was set for multiple reaction monitoring and was operated in a positive-ion mode with an electrospray ionization source. Nitrogen was used as the curtain gas and the collision gas. The ion-spray voltage was set at 5,500 V, and the gas temperature at 500°C. The transition ion pairs were at m/z 325/163 for skimmin, m/z 455.3/160.8 for apiosylskimmin, m/z 163/107 for umbelliferone, and m/z 179/123 for esculetin.

### Acute toxicity assessment for HP oral administration

To assess the acute toxicity of HP by oral administration, a dosage of HP of 5 g/kg was used for the single acute toxicity assay. Female and male C57BL/6 mice were used (*N* = 5 per sex); the same number of mice was used in a vehicle control group. After giving 5 g/kg HP, general symptoms and mortality were observed every hour for 12 h, and continued for the next 14 days. During the experimental period, the mortality and type/degree of symptoms, if any, were recorded for each animal. Every 3 days, the body weight was measured.

On day 14, after overnight fasting, all animals were euthanized, and blood was collected. Immediately after blood collection, the liver, heart, kidneys, lungs, thymus, spleen, and stomach were weighed. Mean organ-to-terminal body weight ratios were calculated using the fasting body weight on the final day. The organs were fixed in 10% formalin. The tissues were embedded in paraffin blocks, sectioned, mounted on slides, stained with hematoxylin and eosin (H&E), and then observed under a microscope (×400).

Serum was used to measure AST (aspartate aminotransferase), ALT (alanine aminotransferase), BUN, albumin, HDL-cholesterol and triglyceride levels using an automated analyzer (200 FR, Toshiba, Tokyo, Japan).

### Prediction analysis of pharmacological targets based on network pharmacology

The four compounds, skimmin, loganin, umbelliferone, and esculetin, were selected for drug-target predictions. Skimmin and loganin are main components of HP, and umbelliferone and esculetin are major metabolites at higher blood drug concentration; their chemical structures are listed in Supplemental Figure 1B. The 3D molecular structures of these four compounds were downloaded from the “Pubmed compound database,” and were placed into the Similarity ensemble approach (SEA) database (http://sea.bkslab.org/) for simulating molecular docking. The predicted targets were input to Therapeutic Target Database (TTD), PharmGKB, and Drugbank databases to identify their roles in inflammation and renal disease.

### Statistical analysis

All measurement data are presented as means ± standard error of the mean (SEM), except that pharmacokinetics data were expressed as means ± standard deviation (SD). Three independent experimental replicates were conducted to strengthen data reliability. The statistical analysis was performed using SPSS version 19.0 for Windows. Comparison of the same parameters among all groups was done using one-way analysis of variance (ANOVA), and the differences between pairs of means were tested, post-hoc, with Tukey's test. Difference in survival rates among the groups was compared using Kaplan-Meier survival analysis. *P*-values of less than 0.05 were considered statistically significant.

## Results

### HP extract shows renal protective effects toward septic AKI and prolongs the survival of septic mice

**Figure 2:**
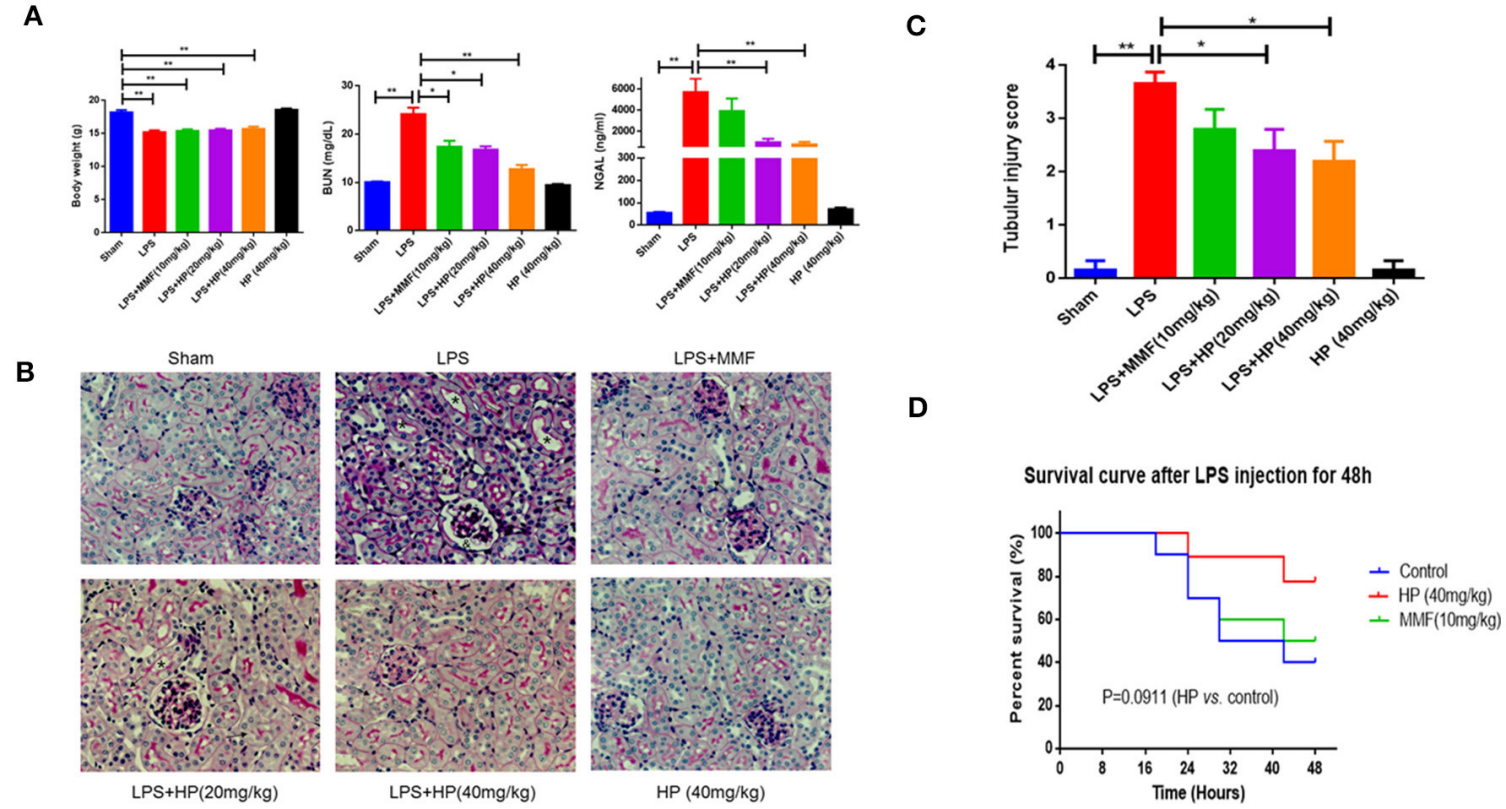
HP alleviates sepsis-induced kidney injury *in vivo*. **(A)** Body weight, the concentrations of plasma BUN and NGAL from different groups, *N* = 8; **(B)** Sepsis-induced histopathologic changes in kidney tissues were detected by H&E staining (magnification 200×). and **(C)** Renal injury score (*N* = 8). **(D)** HP increased the survival of the LPS-induced septic rats with *P* = 0.0911, determined by the Kaplan-Meier method, *N* = 9. ^*^P < 0.05, ^**^P < 0.01.

LPS injection caused serious sepsis in C57BL/6 mice, and the animals showed a sharp drop in body weight and body temperature. Chemical analysis demonstrated that BUN, as well as NGAL, in the plasma was significantly higher compared to that of sham control mice. Meanwhile, HP administration significantly improved renal function by reducing the levels of BUN and NGAL in the plasma in a dose-dependent manner, especially at high doses, which were more effective than MMF at 10 mg/kg (Figure 2A).

Intraperitoneal injection of LPS also caused serious kidney pathological impairments. As observed with PAS staining, we found that LPS caused tissue damage mainly in the renal cortex and outer medulla (Figure 2B). We observed tubular epithelial cell sloughing, loss of brush borders, tubular dilation, and tubular distortion, compared with that of the normal mouse. HP treatment markedly reduced kidney tubular damage (Figure 2B). The quantification of tubular damage confirmed that HP treatment significantly reduced tubular injury in LPS-challenged mice (Figure 2C).

The ability of HP to restore renal function led us to investigate the potential of HP to increase survival following lethal LPS i.p. injection. Three groups of mice were subjected to survival analysis. Drug administration is shown in schematic Figure 1B. Within the 48-h follow-up period, HP improved the mouse survival compared with the survival of the LPS control group, although the difference was not quite significant (Figure 2D; *P* = 0.0911, using the Mantel–Cox log rank test).

### HP reduces oxidative stress in LPS-induced renal tissues

**Figure 3:**
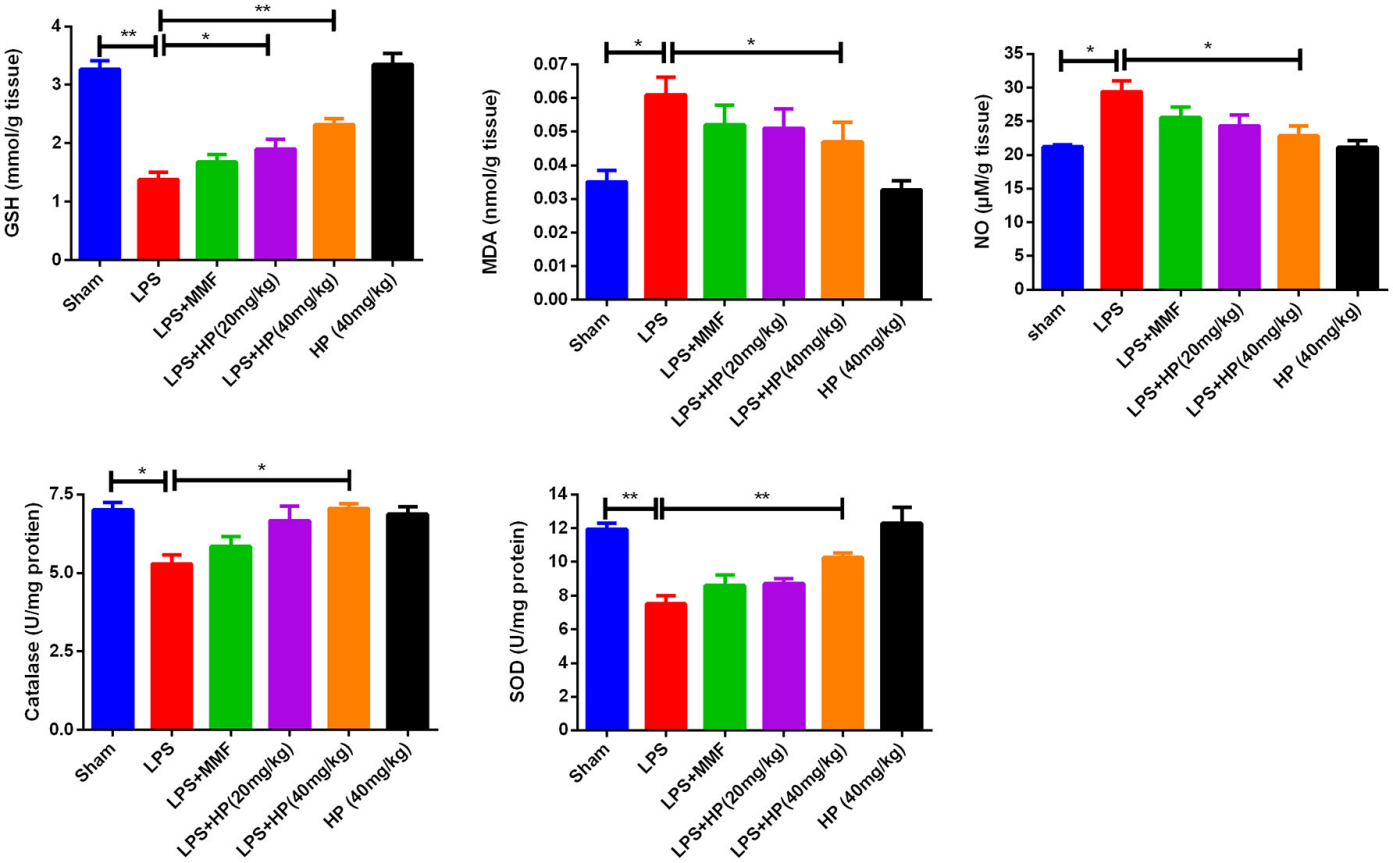
HP dose-dependently ameliorate oxidation stress in kidneys induced by LPS, by reducing MDA and NO production, increasing GSH content in kidney tissues, as well as increasing the activity of SOD and catalase, *N* = 8. ^*^P < 0.05, ^**^P < 0.01.

As shown in Figure 3, LPS i.p. injection augmented oxidative stress in the kidney tissue. NO and malondialdehyde (MDA) levels increased, and GSH, SOD activity, and CAT activity decreased significantly in the LPS-challenged mice compared with the values in the sham mice. HP administration significantly ameliorated the higher oxidative stress by improving the above mentioned five oxidation indexes, especially at higher HP dosages. The antioxidant effect of HP was better than that of MMF at 10 mg/kg.

### HP reduces LPS-induced tubular cell apoptosis by inhibiting the expression and cleavage of caspases

**Figure 4:**
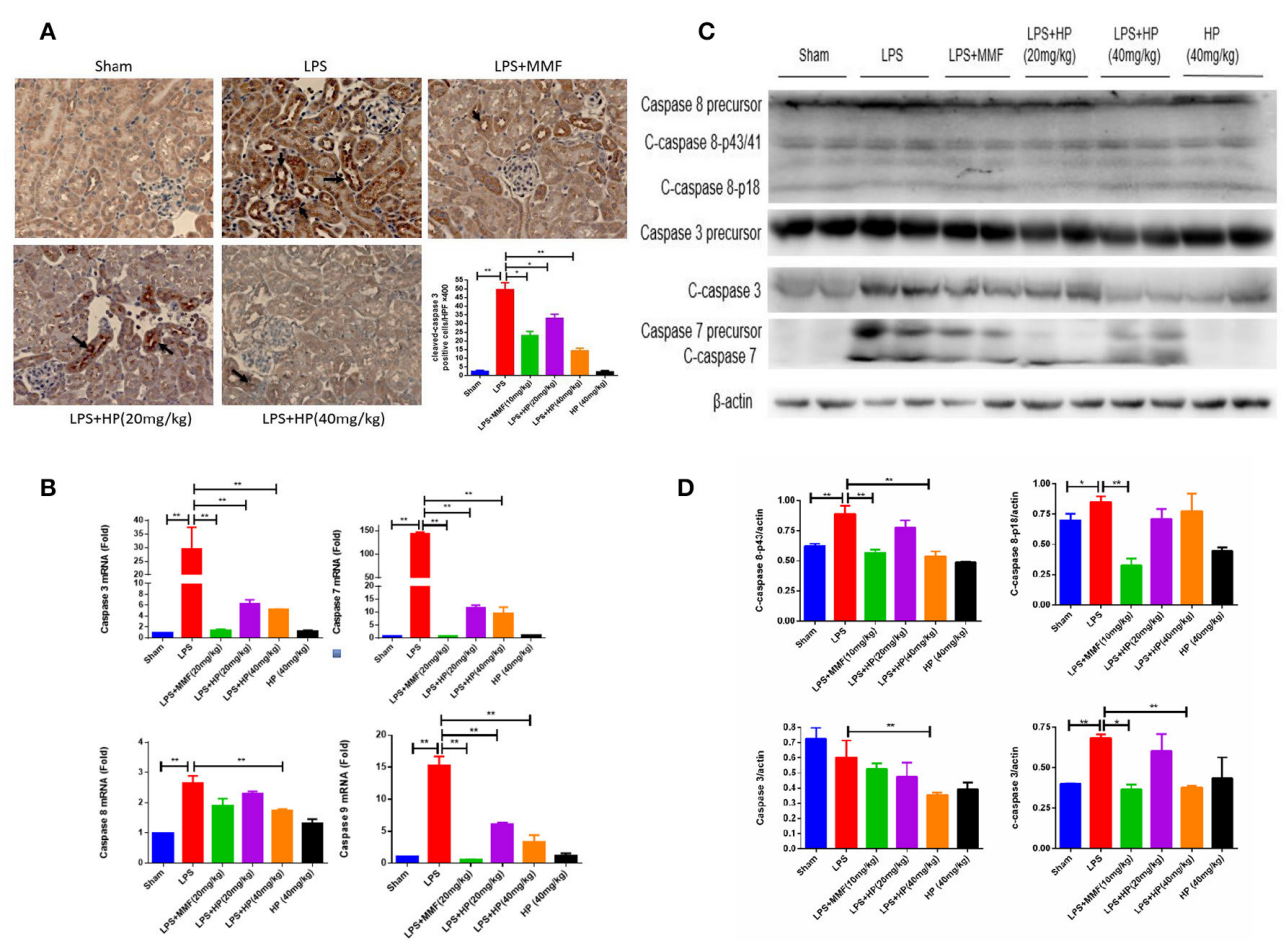
HP significantly reduces the apoptosis of kidney tubular cells induced by LPS. **(A)** Immunohistochemistry of c-caspase 3 in kidney tissue sections; **(B)** HP effect on mRNA levels of caspase 3, 7. 8, and 9 from kidneys by RT-qPCR; **(C)** HP effect on protein levels and cleavage of caspase 3 and caspase 7, and caspase 8 precursor in kidney tissues by western blot and **(D)** quantification of signals and analysis by Image J. *N* = 8. ^*^P < 0.05, ^**^P < 0.01.

As shown in Figure 4A, immunohistochemistry of cleaved caspase 3 (c-caspase 3) showed brown positive staining around tubules in the LPS model group, which is indicative of apoptosis. Meanwhile, HP administration ameliorated the c-caspase 3 staining in a dose-dependent manner. By RT-qPCR, we demonstrated that HP significantly reduced the mRNA levels of caspase 3, 7, 8, and 9, compared with the levels in the LPS model group (Figure 4B). Western blot also indicated that HP treatment could reduce the cleavage of caspase 3 and caspase 7 (Figures 4C,D). Regarding caspase 8, HP significantly suppressed its precursor protein levels compared with those in the LPS model. MMF at 10 mg/kg also ameliorated tubular apoptosis in LPS-induced AKI, but the magnitude of its effect was less than that with HP at 40 mg/kg.

### HP significantly reduces number of monocytes in peripheral blood after stimulated with LPS

**Figure 5:**
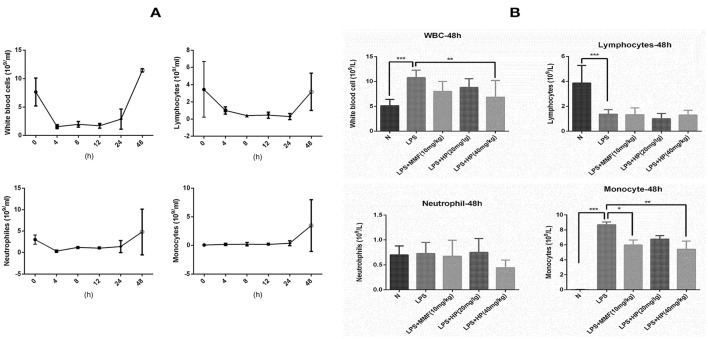
HP significantly inhibits monocyte production *in vivo* by peripheral blood counts after 48 h of LPS i.p. injection. **(A)** Blood cell dynamics of mice injected with LPS within 48 h, including total leukocytes, lymphocytes, neutrophils and macrophages; **(B)** HP effect on blood cell distribution from peripheral blood from different groups at 48 h time point, *N* = 8. ^*^P < 0.05, ^**^P < 0.01, ^***^P < 0.001.

To understand the effect of HP on peripheral blood cells after stimulation with LPS, the dynamic change in peripheral blood leukocyte counts within 48 h of LPS-induced sepsis was first analyzed. As shown in Figure 5A, after LPS i.p. injection, neutrophils and lymphocytes decreased sharply in a short time, which persisted for the first 24 h. At 48 h, the neutrophils and lymphocytes recovered to normal levels. However, although the peripheral blood monocytes showed little change within the first 24 h, they increased sharply by 48 h. HP and MMF administration did not change the peripheral blood counts within the first 24 h (data not shown), but HP and MMF both significantly reduced peripheral blood monocytes by 48 h, which indicates suppressive effects on monocyte production after LPS i.p. injection (Figure 5B).

### HP reduces infiltration of macrophages and neutrophils into kidneys of LPS-induced septic mice

**Figure 6:**
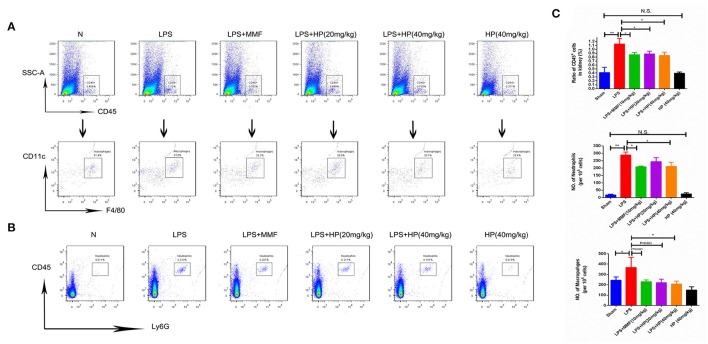
HP significantly reduces the infiltration of neutrophils and macrophages in kidneys of LPS-induced AKI mice. **(A)** Infiltration of CD45^+^, CD11C^+^, and F4/80^+^ macrophages and **(B)** CD45^+^ ly6G^+^ neutrophils in kidneys by flow cytometry; **(C)** quantification analysis of flow cytometry, *N* = 8. ^*^P < 0.05, ^**^P < 0.01.

To further investigate the infiltration of neutrophils and macrophages into the kidney tissues, flow cytometry was used. Single cell suspensions from the kidneys were labeled with mouse CD45, Ly6G, F4/80, or CD11c. As shown in Figure 6, LPS challenge significantly promoted the infiltration of CD45^+^ F4/80^+^ CD11c^+^ macrophages and CD45^+^Ly6G^+^ neutrophils, while HP administration reduced the infiltration of both types of cells; MMF produced similar inhibitory results.

### HP reduces mRNA levels of cytokines and chemokines in LPS-challenged kidney tissues

**Figure 7:**
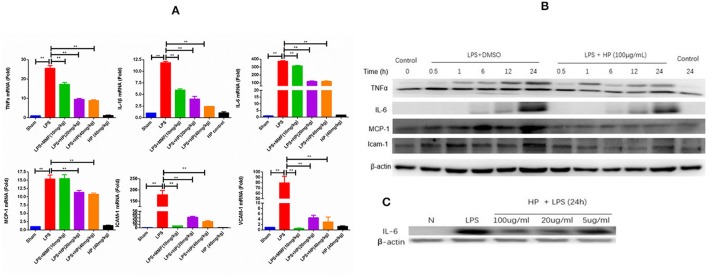
HP significantly inhibits the production of cytokines and chemokines induced by LPS *in vivo* and *in vitro*. **(A)** mRNA expressions of IL-1β, IL-6, TNF-α, MCP-1, ICAM-1, and VCAM-1 from kidneys in LPS-injected mice are significantly increased compared with that of sham controls, and treatment with HP significantly suppressed all increases, *N* = 8. **(B)** Western blot suggests that HP decreases the protein production of TNF-α, MCP-1, and ICAM-1 time-dependently and **(C)** IL-6 dose-dependently in Ana1 cells with LPS stimulation. Each bar represents the mean ± SEM. ^**^P < 0.01.

As shown in Figure 7A, LPS challenge induced the over-production of a series of cytokines and chemokines in AKI kidneys, such as TNFα, IL-1β, IL-6, MCP1, ICAM-1, and VCAM-1, as assessed by RT-qPCR. HP administration significantly reduced the mRNA overexpression of these cytokines and chemokines in the LPS-induced kidney tissues in a dose-dependent manner.

In addition to the *in vivo* study on the anti-inflammatory effects of HP, an *in vitro* study of the HP effect on the expression of cytokines and chemokines in LPS-stimulated Ana1 cells was performed. As shown in Figure 7B, the protein levels of TNF-α, IL-6, MCP1, and ICAM-1 increased in a time-dependent manner due to LPS (50 μg/mL) stimulation in Ana1 cells, and HP reduced their expressions at each time point. However, in our cultured cell system, IL-6 protein levels increased slowly within 24 h, unlike those of TNF-α, MCP1. and ICAM-1. Therefore, to evaluate the effect of HP on IL-6 protein expression, Ana1 cells were stimulated with LPS (50 μg/mL) and co-treated by HP at three concentrations (100, 20, and 5 μg/mL) for 24 h. As shown in Figure 7C, HP significantly inhibited IL-6 protein expression in Ana1 cells stimulated by LPS in a concentration-dependent manner at 24 h.

### HP inhibits activation of the NF-κB signaling pathway both in HK-2 and Ana1 cells

**Figure 8:**
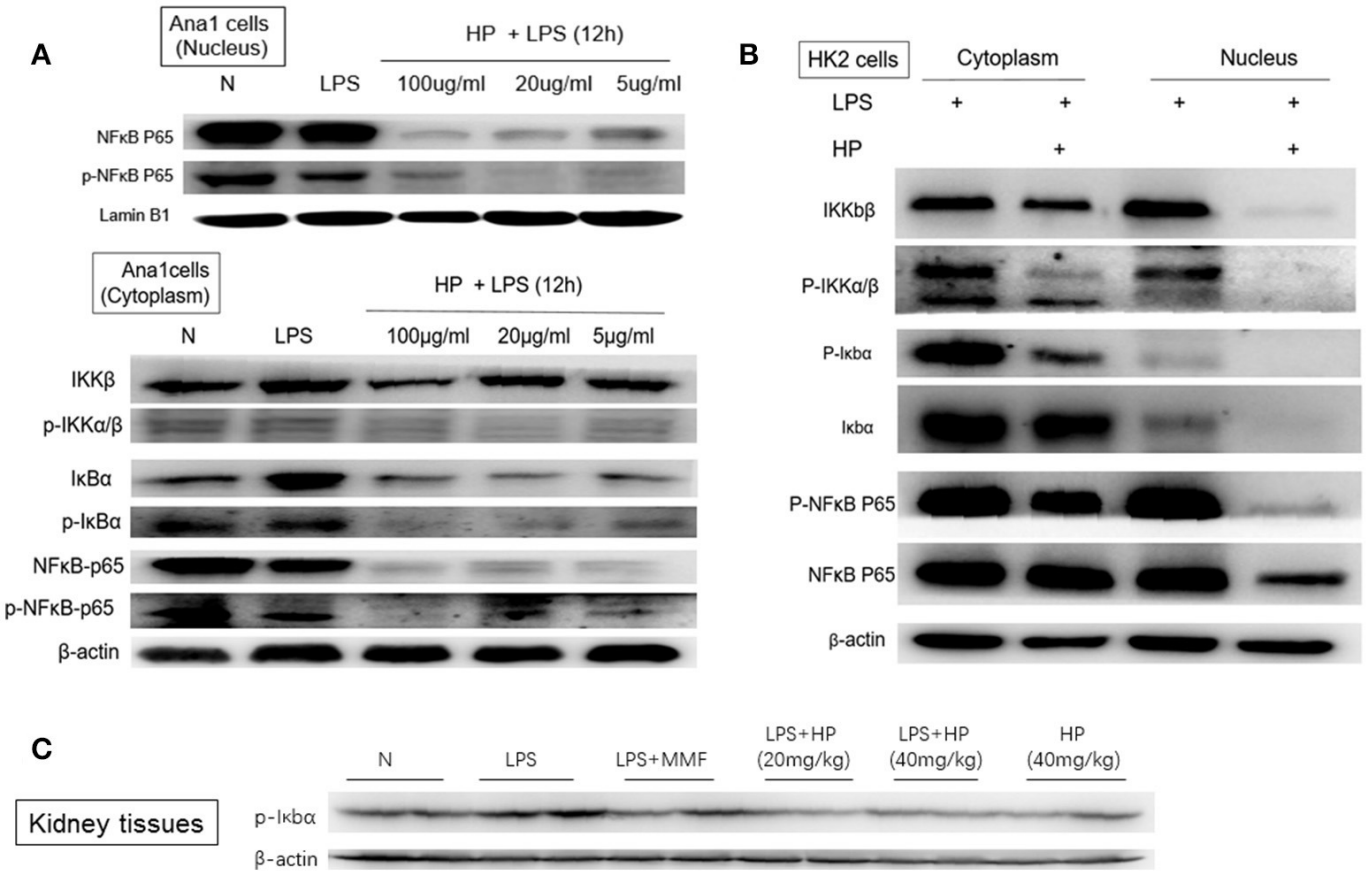
HP blocks LPS-induced NF-κB nuclear translocation *in vitro* and *in vivo* by inhibiting the phosphorylation of IκBα and Iκ*κα*/β in **(A)** Ana1 cells, **(B)** HK-2 cells, and **(C)** kidney tissues.

Using the CCK8 assay, we confirmed non-cytotoxic concentrations of HP for *in vitro* cell experiments. When incubating with HP for 72 h, < 100 μg/mL was not -cytotoxic to Ana1 cells and < 50 μg/mL was not -cytotoxic to HK-2 cells (data shown in Supplemental Table 1). After LPS and HP were co-incubated for 12 h, cell pellets were collected and cytosolic and nuclear proteins were extracted. As shown in Figure 8A, for Ana1 cells, the nuclear protein levels of NF-κB p65 and p-NF-κB were significantly reduced with HP treatment compared to the levels with LPS alone. More surprisingly, in the cytoplasm, except for total IKKβ, total NF-κB and phosphorylated NF-κB, total IκBα and phosphorylated IκBα, as well as phosphorylated IKKα/β were all inhibited by HP treatment in a concentration-dependent manner, even at 5 μg/mL. Regarding HK-2 cells (Figure 8B), HP also significantly inhibited NF-κB-p65 nuclear translocation, and this inhibition also appeared to be caused by inhibition of phosphorylation of IκBα and IKKα/β (Figure 8B). However, as opposed to that in Ana1 cells, HP did not regulate the total protein levels of IκBα and IKKα/β in HK-2 cells; HP only influenced their phosphorylation. Western blots of whole kidney tissue homogenates also demonstrated that HP administration could significantly inhibit phosphorylated IκBα in LPS-challenged kidney tissues (Figure 8C). HP treatment of HK-2 and Ana1 cells for 24 h without LPS stimulation did not influence NF-κB nuclear translocation, or its protein expression in the cytosolic and nuclear extracts (detailed data is shown in Supplemental Figure 2).

### HP inhibits JAK-STAT and MAPK signaling pathways in Ana1 and HK-2 cells

**Figure 9:**
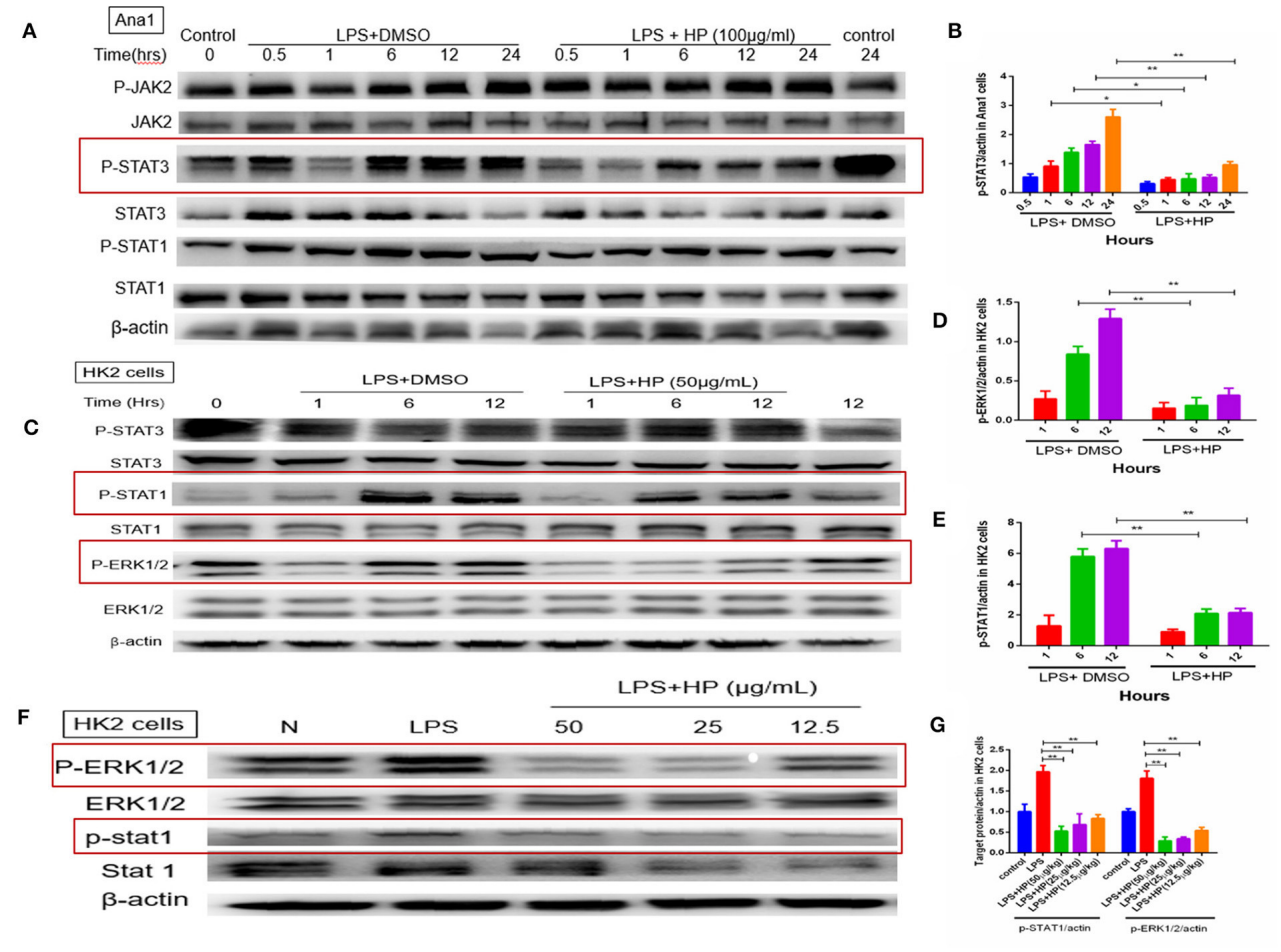
HP significantly inhibits phosphorylation of STAT3, STAT1, and ERK1/2 *in vitro* with LPS stimulation. **(A,B)** HP significantly inhibits STAT3 phosphorylation time-dependently in Ana1 cells stimulated by LPS; **(C–E)** HP significantly inhibits STAT1 and ERK1/2 phosphorylation in HK-2 cells stimulated by LPS time-dependently. **(F,G)** HP significantly inhibits STAT1 and ERK1/2 phosphorylation in HK-2 cells stimulated by LPS dose-dependently. ^*^P < 0.05, ^**^P < 0.01.

As shown in Figures 9A,B, with LPS stimulation *in vitro*, the phosphorylation level of STAT3 in Ana1 cells increased quickly in a time-dependent manner, and HP reduced its phosphorylation at each time point. However, HP did not affect the protein levels of total JAK2 and p-JAK2 in Ana1 cells, which indicates that its inhibitory effect on p-STAT3 might be independent of JAK activity. Meanwhile, HP did not change p-STAT1 in the LPS-stimulated Ana1 cells (Figure 9A). As opposed to that of the macrophagic Ana1 cells, as shown in Figures 9C–G, the phosphorylation of STAT1 and ERK1/2 in LPS-stimulated HK-2 cells was significantly reduced by HP in a time and concentration-dependent manner. However, p-STAT3 was unchanged by HP treatment in LPS-stimulated HK-2 cells.

### Pharmacokinetics study of skimmin, apiosylskimmin, and their predicted metabolites

**Figure 10:**
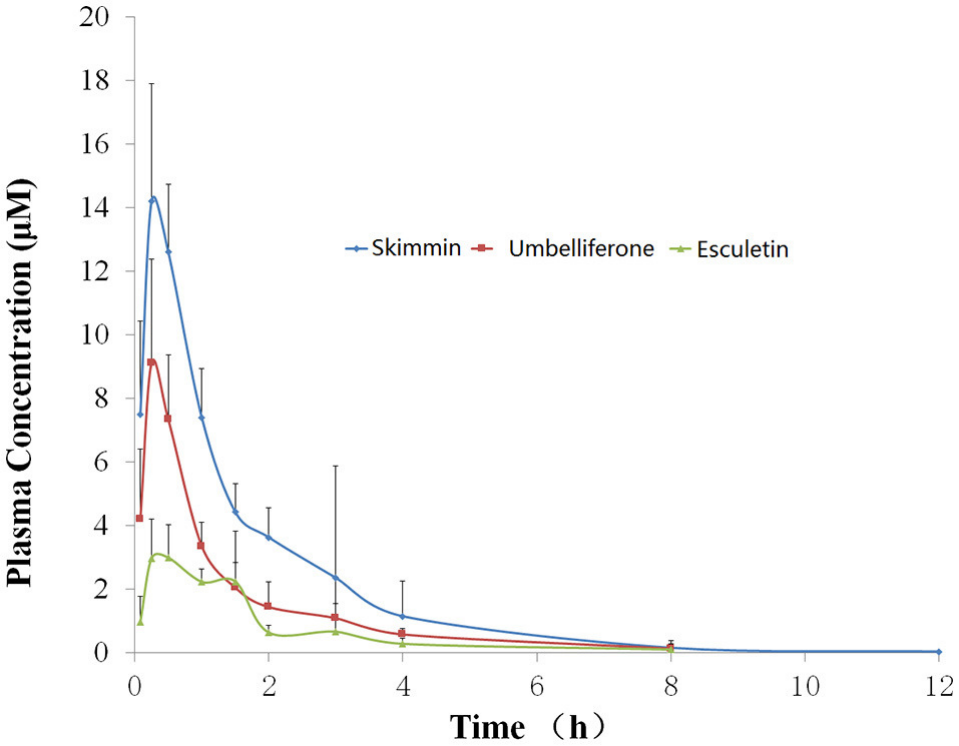
Plasma concentration-time curve of skimmin, umbelliferone, and esculetin in mice following a single oral dose of 50 mg/kg of HP (*n* = 5). Data are expressed as mean ± SD.

As the most abundant coumarin glycoside in HP, the pharmacokinetic profile of plasma skimmin is shown in Figure 10, Tables 1, 2 after a single oral administration of 50 mg/kg in the C57BL/6 mouse. The oral absorbance of skimmin was rapid; it reached its maximum concentration in 0.25 h, with an average plasma drug concentration of 14.21 μM, a mean residence time (MRT (0–t)) of 1.75 h, and an area under the curve (AUC)(0–t) of 23.32 μM/h. Two predicted active metabolites were also found in the plasma at higher blood concentrations. The 7-hydrogen coumarin (umbelliferone) reached its maximum concentration after 0.25 h, with an average blood concentration of 9.12 μM and an AUC (0–t) of 11.83 μM/h. The 6, 7-hydrogen coumarin (esculetin) reached its maximum concentration in 0.5 h, with an average blood concentration of 3.00 μM and an AUC (0–t) of 6.27 μM/h (Figure 10, Tables 2, 3). The concentration of plasma apiosylskimmin was too low to be determined.

**Table 2:** Blood drug concentrations of major coumarins from HP and their predicted metabolites (skimmin, umbelliferone, and esculetin) in mice following a single oral dose of 50 mg/kg at different time points.

**Time (h)**	**Blood drug concentration (**μ**M)**		
	**Skimmin**	**Umbelliferone**	**Esculetin**
0.08	7.50 ± 2.94^*^	4.21 ± 2.21	0.98 ± 0.80
0.25	14.21 ± 3.71	9.12 ± 3.28	2.98 ± 1.24
0.5	12.62 ± 2.12	7.35 ± 2.04	3.00 ± 1.05
1	7.41 ± 1.53	3.36 ± 0.76	2.24 ± 0.40
1.5	4.44 ± 0.89	2.06 ± 0.79	2.25 ± 1.59
2	3.64 ± 0.94	1.45 ± 0.79	0.65 ± 0.22
3	2.37 ± 3.51	1.10 ± 1.30	0.68 ± 0.88
4	1.16 ± 1.11	0.59 ± 0.17	0.30 ± 0.16
8	0.17 ± 0.22	0.14 ± 0.14	0.12 ± 0.01
12	0.05 ± 0.02	Not detectable	Not detectable

* *Values are means ± SD (n = 5)*.

**Table 3:** Pharmacokinetic parameters of major coumarins and their active metabolites (skimmin, umbelliferone, and esculetin) in mice following a single oral dose of 50 mg/kg.

**Index**	**Unit**	**Skimmin**	**Umbelliferone**	**Esculetin**
T_max_	h	0.25	0.25	0.50
C_max_	μM	14.21	9.12	3.00
AUC_(0−t)_	μM/h	23.32	11.83	6.27
AUC_(0−∞)_	μM/h	23.42	12.16	6.66
MRT_(0−t)_	h	1.75	1.58	1.82
MRT_(0−∞)_	h	1.81	1.82	2.38
t_1/2_	h	1.55	1.69	2.30

*AUC_(0–x)_, area under the plasma concentration–time curve from 0 to x h; C_max_, maximum plasma concentration; T_max_, time at maximal concentration; t_1/2_, elimination half-life; MRT_(0–x)_, mean residence time from 0 to x h. Values shown are means (n = 5)*.

### Possible drug targets of major compounds of HP predicted by network pharmacology

**Figure 11:**
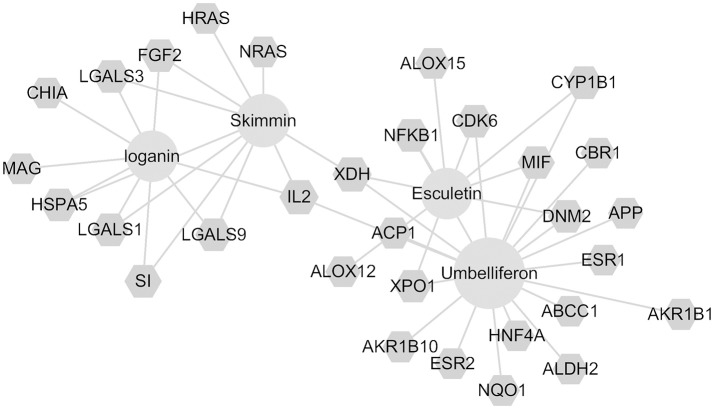
Compound-target network for skimmin, loganin, umbelliferone, and esculetin.

Through the SEA database, a total of 91 predicted human targets were obtained for skimmin, loganin, umbelliferone, and esculetin and 31 inflammation-related targets were screened with additional databases (i.e., RGD, TTD, PharmGKB, and Drugbank). The network graph is shown in Figure 11, which suggests that each compound acts on multiple targets. Umbelliferon was predicted to have the most potential targets; literature retrieval demonstrated that umbelliferon has wide anti-inflammatory and antioxidant bioactivities (Muthu et al., 2015; Sim et al., 2015). Among these possible targets, IL-2, XDH (Xanthine dehydrogenase/oxidase), Cox-2 (Cyclooxygenase-2), and NF-κB are closely related to inflammation. Through interaction of its constituent compounds, HP may synergistically act on different targets, leading to an integrated anti-inflammatory effect.

### HP does not show significant toxic effects in single-dose acute toxicity experiments

Slow movements and huddling of C57BL/6 mice were noticed 2 h after oral administration of HP, but these overt signs of toxicity disappeared soon afterward, with no abnormal signs observed thereafter over the 14-day experiment.

The changes in weight of the mice during the experimental period are shown in Supplemental Figure 3A. In the first 2 days, the body weights of the mice decreased, regardless of sex, but after 14 days, the body weights recovered. After 14 days, the average body weights were higher in the HP-treated mice than in the control mice, although the difference was not significant.

The final organ/body weight ratios are shown in Supplemental Figure 3B, including those of the liver, heart, lungs, kidneys, spleen, thymus, and stomach. No significant changes were observed in any of the relative organ weights between the control group and the treated group.

Biochemical parameters in the serum provide an index of the functional changes in the whole body, helping to determine the presence of infection or disease. AST and ALT are considered specific indicators of hepatocellular necrosis, and BUN and creatinine levels are used as indicators of renal function. There was no significant difference in the AST, ALT, BUN, serum creatinine, albumin, triglyceride, LDL, or HDL between the control group and treated group in the C57BL/6 mice (Supplemental Figure 3C).

Treatment of toxic substances usually induces neutrophilic infiltration and cell death in different organs. Therefore, histopathological observation is important to evaluate the safety of chemical agents. In the current acute toxicity test, seven major organs were collected for H&E staining and representative figures are shown in Supplemental Figure 4; no neutrophilic infiltration or histologic injury was observed in the HP-treated group.

## Discussion

Endotoxin (LPS) administration to experimental animals is frequently used as a surrogate model for human infectious sepsis (Goncalves et al., 2010). In the current study, we used this septic AKI model to explore and finally demonstrate that an extract of *H. paniculata*, a traditional Chinese herb, might have therapeutic potential in LPS-induced AKI.

Serum creatinine in LPS-treated mice does not increase in the current study compared with that in the sham mice, suggesting that serum creatinine is not a good marker for septic AKI, which was also reported in another study (Doi et al., 2009). NGAL is considered a promising biomarker for early diagnosis of AKI (Arena et al., 2010; Han et al., 2012), and its expression level is correlated with the severity of renal impairment during the acute and chronic inflammatory state. In the present study, we confirmed that HP could significantly reduce the increased serum NGAL concentration, as well as the BUN concentration. Histological analysis by PAS-staining also showed that less tubular damage was observed in the HP-treated mice. Both biochemical analyses and histological analyses suggest that HP has renal protective effects on LPS-induced AKI in mice.

The generation of reactive oxygen species (ROS) triggered by LPS plays a key role during the pathogenesis of sepsis (Koyner et al., 2008). The present results clearly indicate the severity of oxidative stress in kidney tissues of LPS-treated mice, which was demonstrated by a significant elevation in LPO content and a significant reduction in GSH content, along with the inhibition of SOD and catalase activities in kidney tissues. HP administration showed an impressive effect on reducing oxidative stress, although the main constituents from HP, such as skimmin, apiosylskimmin, and loganin, do not possess active reducing groups, such as phenolic hydroxyl groups. However, the pharmacokinetics investigation found two metabolites with high blood drug concentrations, namely umbelliferone and esculetin. They have been reported to have strong antioxidant bioactivities, which provides fundamental understanding of the antioxidant effect of HP (Kanimozhi et al., 2011; Garcia-Molina et al., 2013; Subramaniam and Ellis, 2016). The concentration of plasma apiosylskimmin was too low to be determined; we hypothesize that this could be due to rapid hydrolysis by gum bacteria and liver metabolic enzymes.

Although LPS i.p. injection caused a quick reduction in leukocytes, including lymphocytes and neutrophils, in the peripheral blood within a few hours, a rapid infiltration of neutrophils and macrophages was observed in the kidney tissues by flow cytometry. The mechanisms of HP inhibition of leukocyte infiltration may derive from inhibition of chemokines. RT-qPCR of mRNA from kidney tissues and western blots of cell lysates from the macrophage cell line, Ana1, showed that HP could significantly inhibit production of chemokines (e.g., MCP-1, ICAM-1, and VCAM-1) induced by LPS stimulation *in vivo* and *in vitro*. Previous studies have demonstrated that chemokines play important roles in LPS-induced AKI. For example, depleting ICAM-1 can significantly protect renal function (Knotek et al., 2001), and inhibiting MCP-1 can improve renal function in the septic mouse (Deshmane et al., 2009; Lee et al., 2014). Forty-eight hours post-LPS injection, HP at 40 mg/kg significantly decreased monocytes in the peripheral blood, as did MMF, which suggests that the inhibition of the production and infiltration of macrophages is one of the main mechanisms of HP against septic AKI.

Apoptosis plays an important role in LPS-induced AKI. Unlike in ischemia-reperfusion AKI and nephrotic-medication-induced AKI (e.g., with cisplatin), necrosis is not common in septic AKI. PAS staining demonstrated that necrosis was not severe in the present animal model, while the apoptosis indicator, cleaved caspase 3, was significantly activated, as indicated by immunohistochemistry. Another study demonstrated that caspase 3 activity was significantly and positively correlated with plasma creatinine levels in septic AKI patients (Lee et al., 2012). This same study showed that caspase 3 inhibitor pretreatment could inhibit renal tubular cell apoptosis and attenuate kidney dysfunction in septic AKI (Lee et al., 2012). In our current study, HP not only inhibited the mRNA expression of caspases 3, 7, 8, and 9, but also significantly inhibited the cleavage (i.e., activation) of caspase 3 and 7, as assessed by western blot. All of the results suggest that HP can inhibit the expression and activation of caspase family members, thus ameliorating the apoptosis in septic AKI. In Figure 4C of the current study, by western blot, caspase 7 precursor expression in LPS+HP (20 mg/kg) samples was lower than that of LPS+HP (40 mg/kg), which appears contradictory. We have two possible explanations. First, the western blot samples are from animal tissue, and the lower level of caspase 7 precursor may be due to individual variations between mice. Second, inhibition of the caspase 7 precursor may be more sensitive to lower dosages of HP due to unknown mechanisms, which sometimes happens in pharmacological research. In pharmacological animal studies, higher doses do not always produce better results compared to those of lower doses; for some indexes, lower dosages of Traditional Chinese medicine sometimes shows better efficacy.

The major protective effects of HP are linked to its inhibition of LPS-induced kidney inflammation. HP treatment suppressed upregulation of LPS-induced TNF-α, IL-1β, and IL-6 in kidneys *in vivo*. Macrophages and tubular epithelial cells are two types of cells that are impaired in kidneys from LPS-induced AKI. NF-κB activation is regarded as a central event leading to inflammatory in LPS-induced AKI (Liu and Malik, 2006). NF-κB activation requires the phosphorylation of the inhibitory IκB proteins. Our data suggest that HP can inhibit LPS-induced NF-κB activation by downregulating IκBα and IKKα/β phosphorylation in both macrophages and tubular epithelial cells. HP is more effective on macrophage cells, because HP not only inhibited the phosphorylation of IκBα and IKKα/β, but also reduced IκBα levels in Ana1 cells. However, in HK-2 cells, HP only inhibited p-IκBα and p-IKKα/β; the detailed reasons for these differences need further study.

Our *in vitro* study demonstrated that HP could significantly inhibit the phosphorylation of STAT1 and ERK1/2 in LPS-stimulated HK-2 cells. Some studies have reported that STAT1 might play a critical role in innate immunity against Gram-negative bacterial infection, especially for inducible nitric oxide synthase (iNOS) expression (Ohmori and Hamilton, 2001; Stempelj et al., 2007). STAT1 plays a role in Toll-like receptor (TLR) signal transduction and inflammatory responses (Luu et al., 2014), and HP inhibition of NO production in kidney tissues may have been caused by STAT1 phosphorylation inhibition. Another study showed that LPS-stimulated ERK1/2 signaling compromised the integrity of the airway epithelial barrier and initiated the migration of leukocytes across the epithelium, and that epithelial permeability was diminished by ERK1/2 blockade (Serikov et al., 2004). In the septic AKI, LPS could significantly increase ERK1/2 activation, thus leading to tissue damage during AKI. HP could protect the tubular epithelial cells, and reduce lymphocyte infiltration by inhibiting ERK1/2 phosphorylation.

STAT3 can bind to acute phase response elements (APRE) on the promoters of several APR genes, promoting acute inflammatory reactions, and IL-6 is one of the most important STAT3 triggers (Hodge et al., 2005; Nechemia-Arbely et al., 2008; Greenhill et al., 2011). Mortality in sepsis has been reported to correlate with the upregulation of IL-6 (Wang et al., 2009). The role of IL-6 as a proinflammatory cytokine in kidney injury has been well-established, and STAT3 is a major downstream signaling target of IL-6 (Yuan et al., 2011). Our results suggest that HP can modulate inflammatory signaling through STAT3 in LPS-induced AKI by inhibiting IL-6-STAT3 activation.

Network pharmacology analysis suggests that the four most abundant compounds from HP interact with 31 inflammation-related targets. Some targets, such as NF-κB, have been indicated by other assays in the current study, while some potential targets, such as Cox-2 and IL-2, will require further study to verify whether they are true targets for the HP constituents. XDH, IL-2, and ACP1 are common targets of the four compounds, which may indicate synergistic activities of the components of HP.

The safety of the HP extract was tested by a single acute toxicity assay in the current study, and we showed that a dosage of even 5 g/kg is relatively safe for the mice; this dosage is much higher than the experimental dosage. However, our preliminary data suggest that dosages above 40 mg/kg are not more efficacious (data not shown), which is why we selected 20 mg/kg and 40 mg/kg in the current study.

Previously, our laboratory had demonstrated that HP could protect against cisplatin-induced acute kidney damage in mice by suppressing renal inflammation and apoptosis (Sen et al., 2017). In the current study, a different AKI model has been used, and deep molecular mechanisms have been investigated and pharmacokinetics has also been performed. To the best of our knowledge, this is the first time that the anti-inflammation-related signaling pathways of *H. paniculata* extract in response to LPS challenge have been elucidated, and a network pharmacological analysis for its major bioactive components has been performed. These thorough studies help us understand more clearly its pharmacological activity, both as a whole extract and as its individual components. Using two animal models, the renal protective effects of HP have been confirmed, which provides greater confidence to advance it into clinical studies in the future.

## Conclusions

In summary, considering all abovementioned data, HP has satisfactory renal protective effects on LPS-induced septic AKI. HP also has good safety and oral bioavailability. Therefore, it has potential to be used in clinical trials for septic patients.

## Availability of data and material

The datasets used and/or analyzed during the current study are available from the corresponding author on reasonable request.

## Author contributions

SZ was in charge of most experiments of the current study and manuscript preparation; JM, JY, and DZ were responsible for HP preparation; LS and DW were responsible for the pharmacodynamics study; JY was responsible for network pharmacology analysis; XC was responsible for the experimental design and funding support.

### Conflict of interest statement

The authors declare that the research was conducted in the absence of any commercial or financial relationships that could be construed as a potential conflict of interest.

## Supplementary material

Supplementary Table 1 Cytotoxicity of HP on HK2 and Ana1 (CCK8 assay,72h).

Supplemental Figure 1 **(A)** Chemical structure of skimmin and apiosylskimmin and HPLC profile of major coumarins from HP. Standard skimmin and apiosylskimmin were purchased from Guilin Huiang Biochemistry Pharmaceutical Company Ltd. (China); **(B)** chemical structures of loganin, umbelliferone, and esculetin.

Supplemental Figure 2 HP does not influence NF-κB nuclear translocation without LPS stimulation in HK-2 cells and Ana1 cells.

Supplemental Figure 3 HP does not show noticeable toxic effects with a 5 g/kg single bolus in an acute toxicity test. **(A)** Animal body weights within 14 days of HP injection; **(B)** Relative organ ratios for several important organs; **(C)** Biochemical test for BUN, AST, ALT, and other indexes potentially influenced by HP, *N* = 5.

Supplemental Figure 4 Eight tissues (including heart, lung, liver, stomach, kidney, thymus, and spleen) stained with hematoxylin and eosin (H& E, 400×) showing the effect of HP in mice. The samples (A) through (D) are from male, and samples (E) through (H) are from female. The oral administration dosage was 5 g/kg, *N* = 5.

## Abbreviations

HP: *Hydrangea paniculata*
AKI: acute kidney injury
BUN: blood urea nitrogen
NGAL: neutrophil gelatinase-associated lipocalin
ICU: intensive care unit
CMC-Na: sodium carboxymethyl cellulose
MMF: Mycophenolate mofetil
LPS: lipopolysaccharide
BCA: bicinchoninic acid
NO: nitric oxide
GSH: reduced glutathione
LPO: lipid peroxidation
SOD: superoxide dismutase
CAT: catalase
SDS: sodium dodecyl sulfate
AST: aspartate aminotransferase
ALT: alanine aminotransferase
ROS: reactive oxygen species
APRE: acute phase response elements
STAT: signal transducer and activator of transcription.
