# Supplementary material for: Total Coumarins from Hydrangea paniculata Show Renal Protective Effects in Lipopolysaccharide-Induced Acute Kidney Injury via Anti-inflammatory and Antioxidant Activities
Source: Front Pharmacol. 2017 Dec 14;8:872. doi: 10.3389/fphar.2017.00872 (PMC5735979; doi:10.3389/fphar.2017.00872)
Supplement: Supplementary Table 1 — Cytotoxicity of HP on HK2 and Ana1 (CCK8 assay,72h). [file Table1.DOCX]

Supplemental table 1 Cytotoxicity of HP on HK2 and Ana1(CCK8 assay，72h)

|  |  | Inhibition（%） | |
| --- | --- | --- | --- |
| compounds | Concentration  （μg/m） | HK2 | Ana1 |
| HP | 100 | 24.9 | 0 |
|  | 50 | 0 | 0 |
|  | 25 | 0 | 0 |
|  | 12.5 | 0 | 0 |
