# Supplementary figures and images for: Total Coumarins from Hydrangea paniculata Show Renal Protective Effects in Lipopolysaccharide-Induced Acute Kidney Injury via Anti-inflammatory and Antioxidant Activities
Source: Front Pharmacol. 2017 Dec 14;8:872. doi: 10.3389/fphar.2017.00872 (PMC5735979; doi:10.3389/fphar.2017.00872)

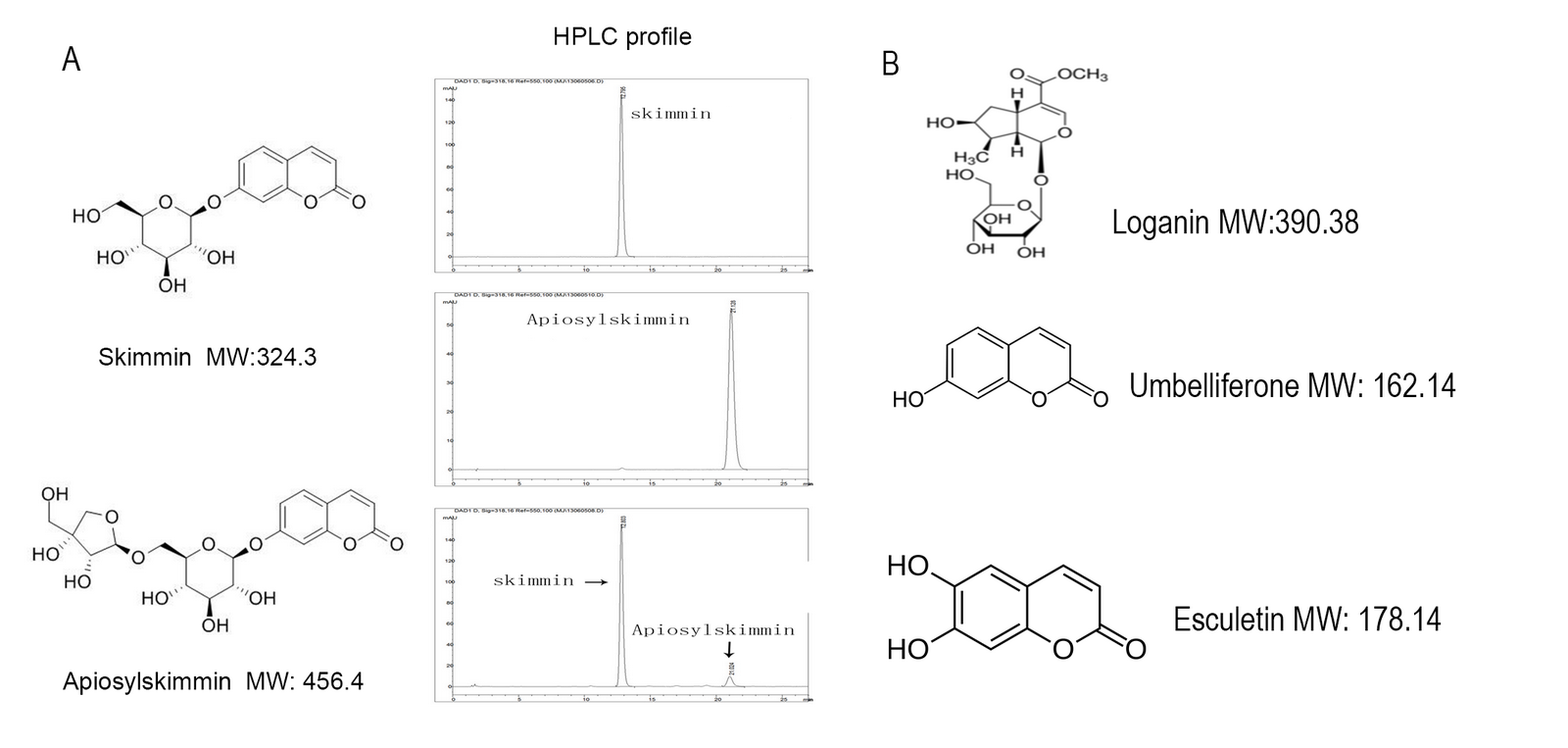

Supplement: Supplemental Figure 1 — (A) Chemical structure of skimmin and apiosylskimmin and HPLC profile of major coumarins from HP. Standard skimmin and apiosylskimmin were purchased from Guilin Huiang Biochemistry Pharmaceutical Company Ltd. (China); (B) chemical structures of loganin, umbelliferone, and esculetin. [file Image1.TIF]

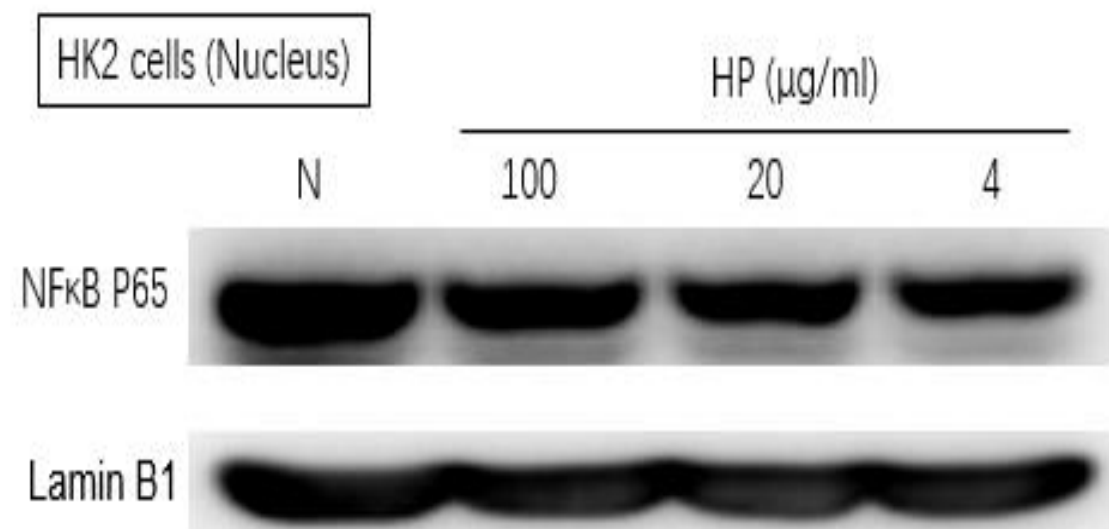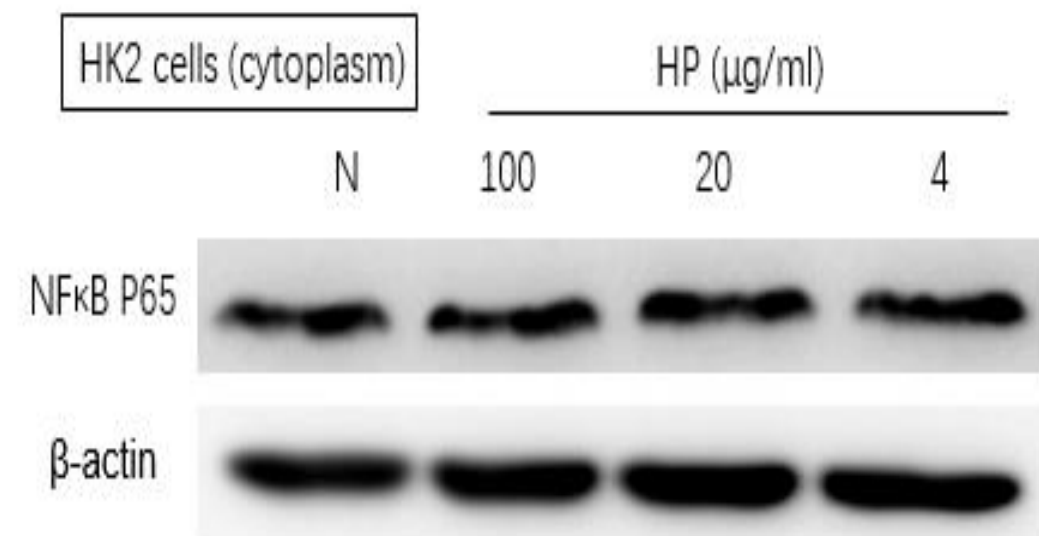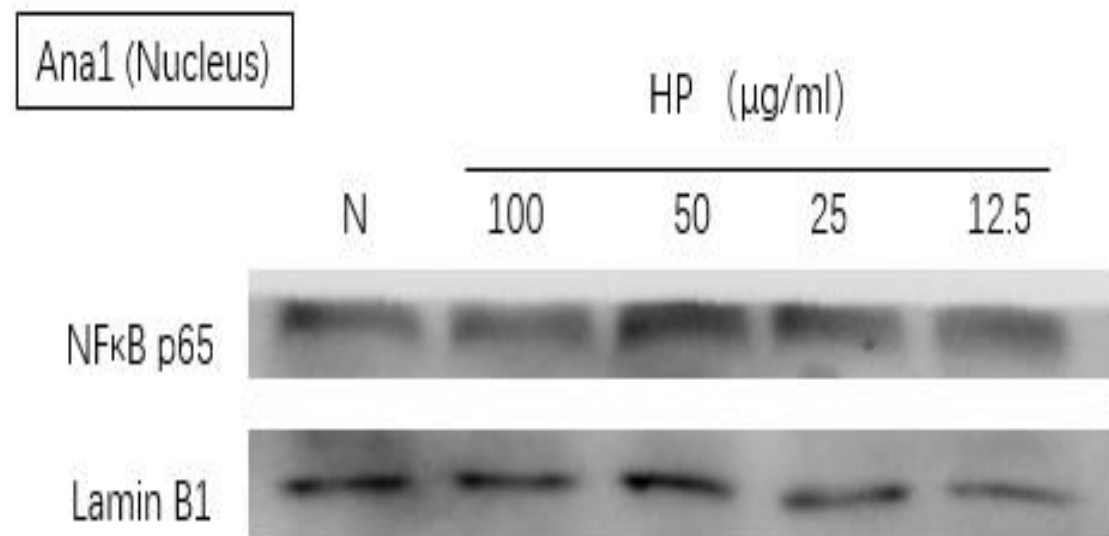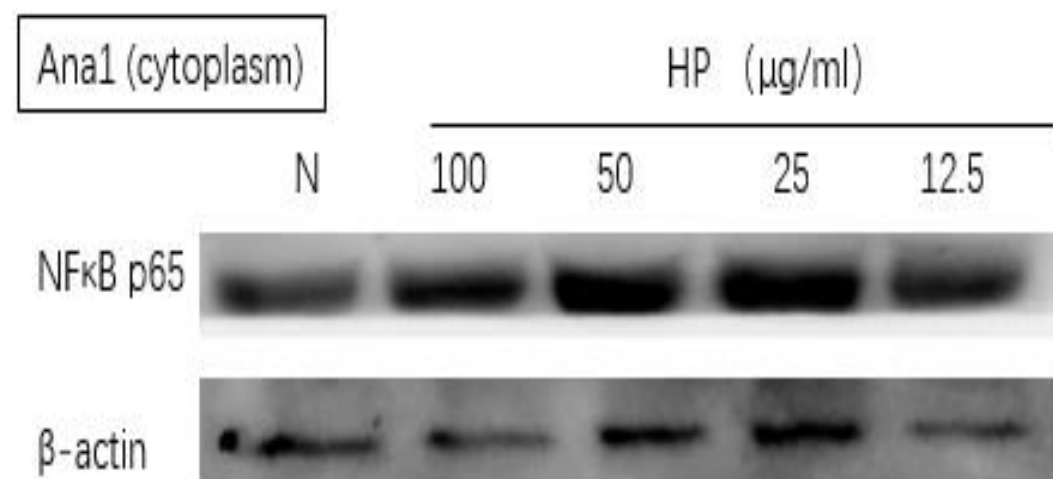

Supplement: Supplemental Figure 2 — HP does not influence NF-κB nuclear translocation without LPS stimulation in HK-2 cells and Ana1 cells. [file Image2.PDF]

A

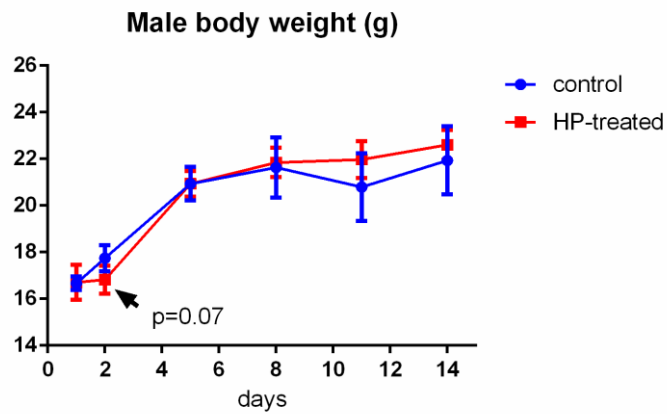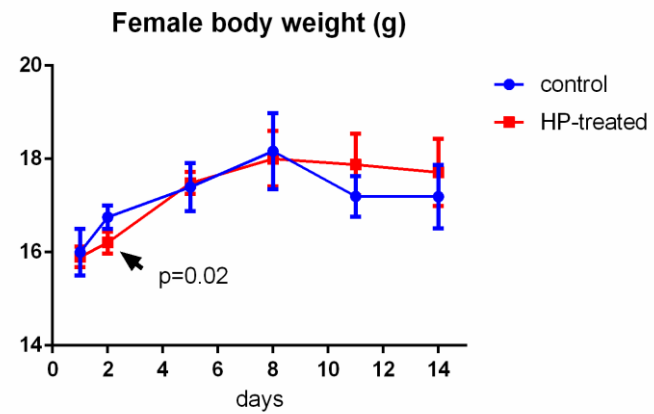

B

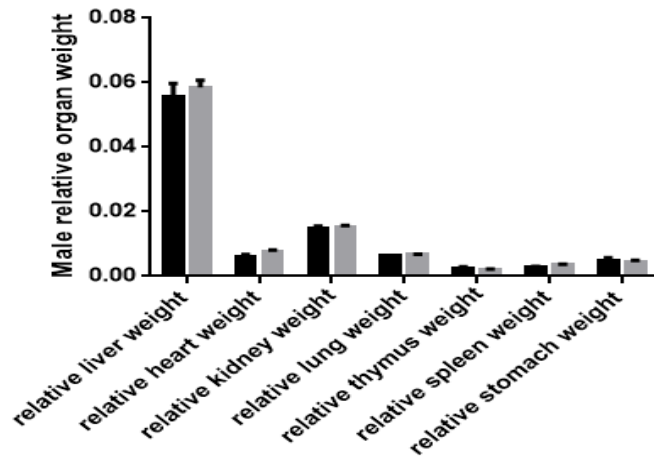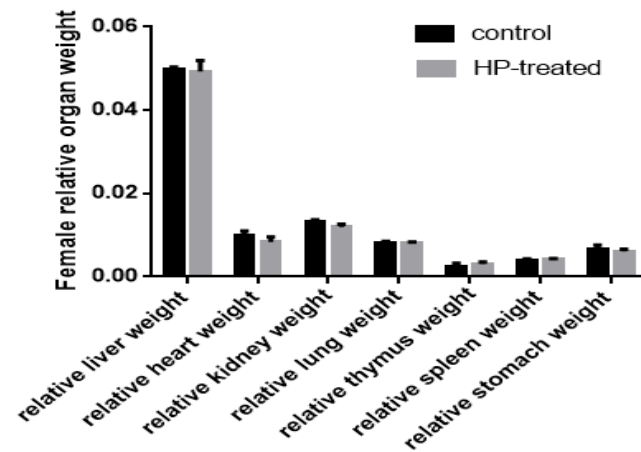

C

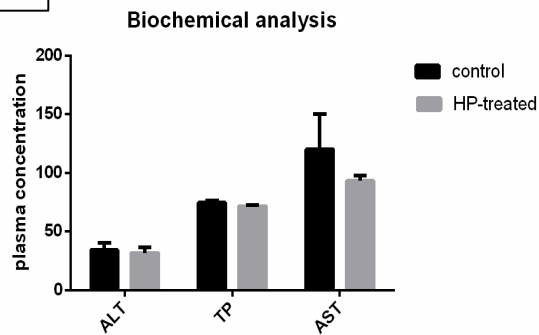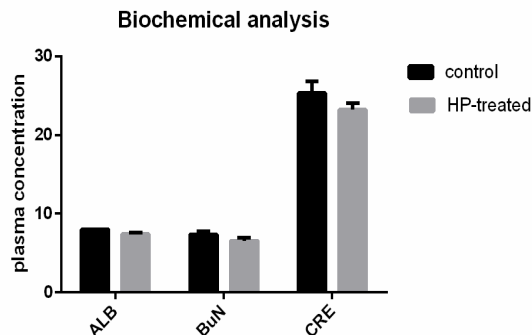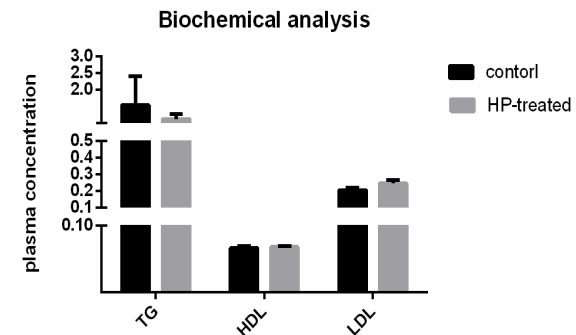

Supplement: Supplemental Figure 3 — HP does not show noticeable toxic effects with a 5 g/kg single bolus in an acute toxicity test. (A) Animal body weights within 14 days of HP injection; (B) Relative organ ratios for several important organs; (C) Biochemical test for BUN, AST, ALT, and other indexes potentially influenced by HP, N = 5. [file Image3.PDF]

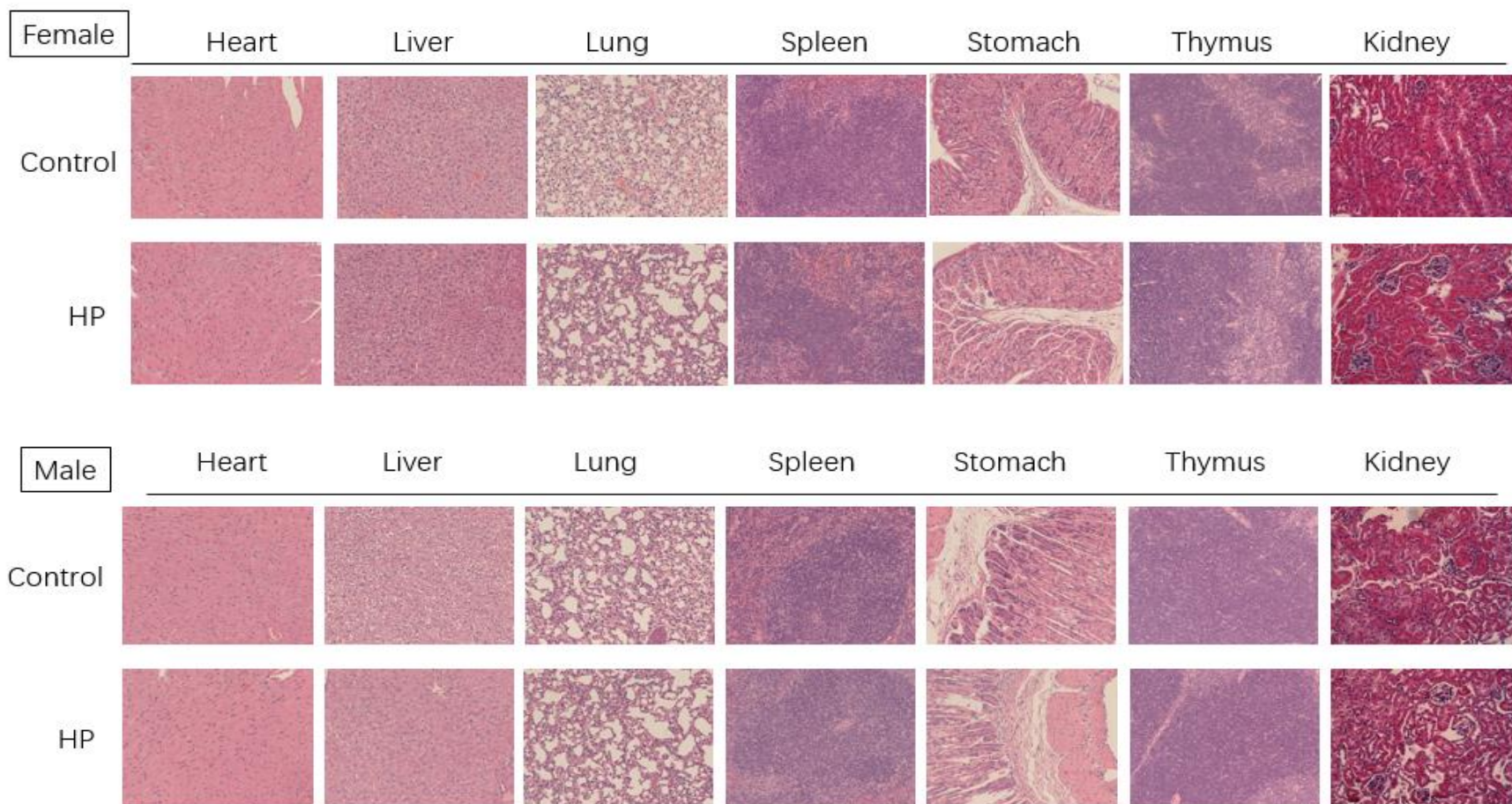

Supplement: Supplemental Figure 4 — Eight tissues (including heart, lung, liver, stomach, kidney, thymus, and spleen) stained with hematoxylin and eosin (H& E, 400×) showing the effect of HP in mice. The samples (A) through (D) are from male, and samples (E) through (H) are from female. The oral administration dosage was 5 g/kg, N = 5. [file Image4.PDF]
